# Exploratory study for clinical signs of MODIC changes in patients with low-back pain in the Netherlands armed forces

**DOI:** 10.1186/s12998-018-0229-4

**Published:** 2019-02-14

**Authors:** Peter van der Wurff, Tom Vredeveld, Caroline van de Graaf, Rikke K. Jensen, Tue S. Jensen

**Affiliations:** 1 Research and Development, Military Rehabilitation Centre Aardenburg, Doorn, The Netherlands; 2 Institute for Human Movement Sciences, HU University of Applied Sciences Utrecht, Utrecht, The Netherlands; 3 School of Physiotherapy, Faculty of Heath, Amsterdam University of Applied Sciences, Amsterdam, the Netherlands; 4 Nordic Institute of Chiropractic and Clinical Biomechanics, Odense, Denmark; 5 Department of Diagnostic Imaging, Silkeborg Regional Hospital, Silkeborg, Denmark

**Keywords:** Modic changes, Clinical tests, Diagnostic value, Magnetic resonance imaging, Low-back pain

## Abstract

**Background:**

Magnetic resonance imaging (MRI) is being used extensively in the search for pathoanatomical factors contributing to low back pain (LBP) such as Modic changes (MC). However, it remains unclear whether clinical findings can identify patients with MC. The purpose of this explorative study was to assess the predictive value of six clinical tests and three questionnaires commonly used with patients with low-back pain (LBP) on the presence of Modic changes (MC).

**Methods:**

A retrospective cohort study was performed using data from Dutch military personnel in the period between April 2013 and July 2016. Questionnaires included the Roland Morris Disability Questionnaire, Numeric Pain Rating Scale, and Pain Self-Efficacy Questionnaire. The clinical examination included (i) range of motion, (ii) presence of pain during flexion and extension, (iii) Prone Instability Test, and (iv) straight leg raise. Backward stepwise regression was used to estimate predictive value for the presence of MC and the type of MC. The exploration of clinical tests was performed by univariable logistic regression models.

**Results:**

Two hundred eighty-six patients were allocated for the study, and 112 cases with medical records and MRI scans were available; 60 cases with MC and 52 without MC. Age was significantly higher in the MC group. The univariate regression analysis showed a significantly increased odds ratio for pain during flexion movement (2.57 [95% confidence interval (CI): 1.08–6.08]) in the group with MC. Multivariable logistic regression of all clinical symptoms and signs showed no significant association for any of the variables. The diagnostic value of the clinical tests expressed by sensitivity, specificity, positive predictive, and negative predictive values showed, for all the combinations, a low area under the curve (AUC) score, ranging from 0.41 to 0.53. Single-test sensitivity was the highest for pain in flexion: 60% (95% CI: 48.3–70.4).

**Conclusion:**

No model to predict the presence of MC, based on clinical tests, could be demonstrated. It is therefore not likely that LBP patients with MC are very different from other LBP patients and that they form a specific subgroup. However, the study only explored a limited number of clinical findings and it is possible that larger samples allowing for more variables would conclude differently.

## Background

Magnetic resonance imaging (MRI) is being used extensively in the management and diagnosis of patients with nonspecific low-back pain (LBP). MRI findings in the lumbar spine e.g. disc and facet joint degeneration have been reported to be more prevalent in people with LBP than in people without LBP [1, 2].

One of the MRI findings that has previously been suggested to be associated with LBP is the presence of Modic changes (MC) [1, 3]. However, a recent systematic review investigating the association between LBP and MC [4] found that the association was inconsistent and that this could to some extent be explained by high risk of bias of the studies and heterogeneity of the study samples included. Therefore, it still remains unclear if patients with MC represents a specific subgroup of patients with LBP. If patients with MC on MRI have a specific clinical pattern that could be identified by clinical findings and questionnaires, MC would represent a specific subgroup that may require different health care strategies.

MC are vertebral endplate and subchondral bone marrow changes that are most readily demonstrated on MRI [4]. Three types of MC have been described. Type 1 (MC1) is characterized by a low intensity on T1 and high-intensity signals on T2-weighted images, whereas Type 2 (MC2) shows high intensity on T1 and isointensity to high-intensity signals on T2-weighted images. Both T1 and T2 show low-intensity signals in cases of Type 3 (MC3), which is seldom seen in patients with LBP [3].

Based on histological studies, MC1 is often described as a form of inflammation starting from the fissured endplates of the lumbar discs merging with vascular granulation tissue into the vertebral body [5]. It is characterized by bone edema appearing in the vertebral body adjacent to the involved endplate. The inflammation theory of MC1 proposes that it could be a result of disc degeneration and new capillarization or maybe inflammation with anaerobic bacteria [6]. In MC2, fatty bone replacement is seen in the bone marrow of the vertebral body, whereas MC3 shows signs of bone sclerosis in the vertebral bodies and endplates [5]. MC3 is, however, rare in adult populations and therefore not considered clinically relevant [3].

A number of studies have reported that certain demographic, clinical, and imaging characteristics increase the probability of having MC. The following demographic and clinical characteristics have been reported to be associated with people having MC, compared to those without: age [7, 8], smoking [9], high Body Mass Index [10], and heavy work load [9]; for demographics and clinical characteristics: pain duration [11], pain intensity [11–13], pain on movement [9], pain with extension [11], inflammatory pain pattern [13], and high-sensitivity, C-reactive protein [13]. In relation to imaging characteristics, disc degeneration [6, 14] and disc herniation [12, 14] increase the probability of people having MC. In addition, two studies have reported that translational instability seems to be associated with LBP and the presence of MC, which may be due to the strong association between disc degeneration and segmental instability [15, 16].

If clinical tests could indicate the presence of MC, it would be more likely that patients with MC form a specific subgroup of LBP. Also, a clear clinical profile would provide quicker and more cost-effective diagnoses and, maybe, even enable early intervention. From a theoretical point of view, quick diagnosis would help patients with a combination of LBP and MC. The prognostic value of MC is not fully understood and the literature shows conflicting evidence. The presence of MC has been found to be both a positive and a negative prognostic factor depending on the type of treatment [17–21]. This is particularly interesting in the military service, where daily physical demands are exceptional due to marching, climbing, and jumping from heights. This could result in adaptation of training or, ultimately, in finding another job*.*

These characteristics raise the question of whether demographic determinants, possibly combined with clinical signs and symptoms, can identify patients who have MC. Therefore, the aim of this explorative study was to assess the predictive value of clinical tests and questionnaires to detect MC in a population of active servicemen of the Netherlands Armed Forces with persistent LBP.

## Methods

### Study design

This is an explorative retrospective cohort study. This manuscript is written according to Strengthening the Reporting of Observational Studies in Epidemiology (STROBE) guidelines [22].

### Setting

The study was conducted at the National Military Rehabilitation Center Aardenburg (MRC), Doorn, the Netherlands, which receives servicemen referred by a military general practitioner or medical specialist, and, occasionally, civilians referred by a general practitioner or medical specialist. Patients are treated with a multidisciplinary approach in an outpatient setting.

### Participants

Patients were included in the study if they had persistent LBP with or without leg pain for a period of three months or longer and were on active duty in the Netherlands Armed Forces. Participants were excluded from the study when serious pathology, i.e., radicular pain with neurological signs, neurogenic claudication, ankylosing spondylitis, tumors of the spine, infections, osteoporosis or recent fractures, was diagnosed. Other reasons for exclusion were, if they were civilians, the presence of urinary or bowel incontinence, previous fractures, psychiatric disorders, and pregnancy.

### Variables and levels of measurement

Data were extracted from medical files and narrative radiology reports [23] or MR-images directly, if available, from patients with persistent LBP referred in the period between April 2013 and July 2016.

The Roland Morris Disability Questionnaire (RMDQ) was used to measure the level of disability, with scores ranging from 0 (none) to 24 (high disability). The Dutch RMDQ has previously shown good reliability (Interclass Correlation Coefficient [ICC]: 0.91) and validity [24]. To assess the perceived level of general pain, the Numeric Pain Rating Scale (NPRS) for LBP was used, on an 11-point scale (0: no pain, 10: indicating severe pain). Research has shown high validity and reliability coefficients for the NPRS [25]. The Pain Self-Efficacy Questionnaire (PSEQ) was used to evaluate pain coping strategies. The PSEQ has a score ranging from 0 (no confidence in situations) to 60 (full confidence in situations) and has shown high reliability and validity [26]. Patient characteristics were collected at admission to the Military Rehabilitation Centre Aardenburg (MRC and included age, gender, and military rank.

Clinical signs and symptoms were tested by protocol of the MRC by a manual therapist during the first visit to the MRC, including flexion and extension range of motion (ROM) and pain patterns. Physical assessment included the Prone Instability Test (PIT) and straight leg raise (SLR). The PIT shows acceptable reliability for testing lumbar instability, with kappa values ranging from 0.46 to 0.87, and good validity (sensitivity: 0.71, specificity: 0.57) The straight leg raise is used to assess the extensibility of the hamstrings [27].

In the MRC, the majority of LBP patients are not routinely scheduled for an MRI scan to evaluate the presence of MC, but rather to assess clinical signs of nerve root compression, disc herniation, and spinal stenosis, or to exclude serious pathology, i.e., cancer, fracture, infection, and systemic disease [28]. The MRI system was a 0.2 T device, and the imaging protocol consisted of sagittal and axial T1- and T2-weighted sequences.

### Statistical methods

Comparison between MC1 and MC2 characteristics was computed with an independent T-test and the Mann Whitney U test. The exploration of clinical tests was performed by univariable logistic regression models. The variables included were flexion and extension range of motion and pain patterns, PIT and SLR, and the NPRS, RMDQ, and PSEQ questionnaires. In cases with a meaningful difference, adjusting analyses were performed.

Secondary analysis consisted of measures of diagnostic accuracy, including sensitivity, specificity, positive predication value (PPV), negative predication value (NPV), positive likelihood ratio, and negative likelihood ratio for each clinical test or questionnaire. Afterward, we assembled all clinical tests and questionnaires into the so-called multiple tests regimens*.* In cases of more than 5% missing values, based on the total data set, multiple imputations were used. Statistical significance was assumed at the a-level of 0.05. All statistics were performed using SPSS 24.0 (IBM Corp 2016, Armonk, NY.)

## Results

**Fig. 1:**
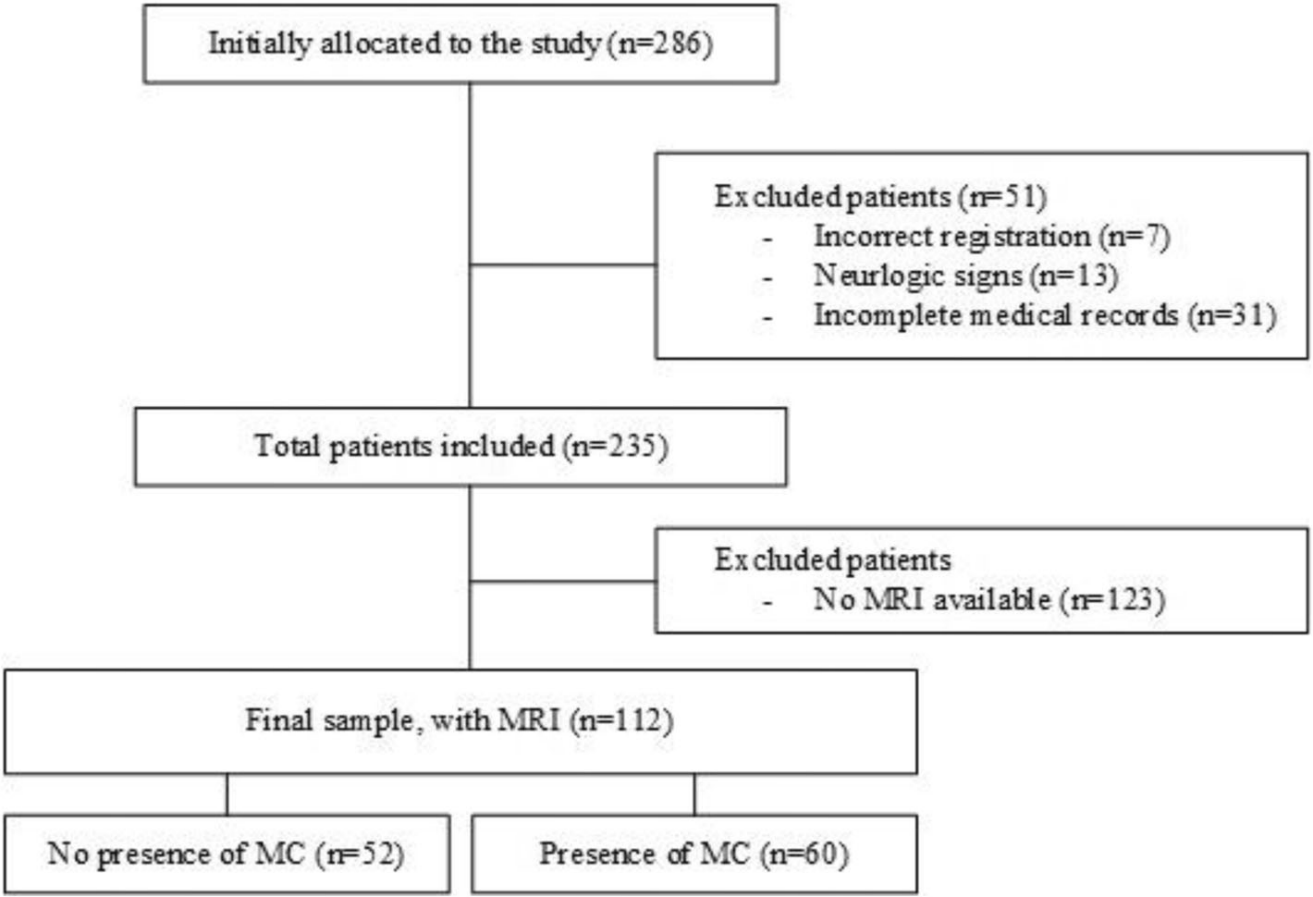
Flow chart of inclusion of participants

In the period between April 2013 and July 2016, a total of 286 patients were referred to the MRC for the treatment of LBP, and 112 of those were included in this retrospective study. Figure 1 shows a flow chart of the inclusion and exclusion of patients.

Of the 112 patients included in the study sample, 101 (90.2%) were male. Age differed significantly, with a median age of 44 in patients with MC and 32 in those without MC (*p* < 0.01). Characteristics of the two groups (MC and no MC) are presented in Table 1. Our data set demonstrated a total of 2% of missing values; therefore, multiple imputations were not needed.

**Table 1:** Characteristics of the participants

	Cases			Total
	MC *n* = 60 (53.6%)	No MC *n* = 52 (46.4%)	*p*-value	*N* = 112 (100%)
Age [median (min–max)]	44 (20–56)	32 (21–62)	*p* < 0.01	36 (20–61)
Gender *n* (%)			*p* = 0.95	
Male	54 (90.0%)	47 (90.4%)		101 (90.2%)
Female	6 (10.0%)	5 (9.6%)		11 (9.8%)
Duration of complaints in months [median (min–max)]	24 (3–192)	35 (2–180)	*p* = 0.85	24 (2–192)
MC type *n* (%)				
Type 0	–	52 (22.1%)		52 (46.4%)
Type 1	33 (14.0%)	–		33 (29.5%)
Type 2	27 (11.5%)	–		27 (24.1%)
Type 3	0 (0%)	–		0 (0%)
Military rank [*n* (%)]			*p* = 0.77	
Enlisted personnel	17 (28.3%)	18 (34.6%)		35 (31.3%)
Noncommissioned officers	33 (55.0%)	26 (50.0%)		59 (52.7%)
Commissioned officers	19 (16.7%)	8 (15.4%)		18 (16.1%)
Positive PIT	45 (75.0%)	32 (61.5%)	*p* = 0.13	77 (68.8%)
Positive SLR	12 (20.0%)	15 (28.8%)	*p* = 0.32	27 (24.1%)
Pain in flexion	49 (81.7%)	33 (63.5%)	p = 0.03*	82 (73.2%)
Pain in extension	46 (76.7%)	33 (63.5%)	p = 0.13	79 (70.5%)
Limited ROM flexion	49 (81.7%)	38 (73.1%)	*p* = 0.28	87 (77.7%)
Limited ROM extension	49 (81.7%)	35 (67.3%)	*p* = 0.08	84 (75.0%)
RMDQ	x̄: 10.97 (sd: 5.36)	x̄: 11.58 (sd: 5.64)	*p* = 0.56	x̄: 11.25 (sd: 5.47)
PSEQ	x̄: 42.70 (sd: 10.24)	x̄: 38.42 (sd: 11.73)	*p* = 0.04*	x̄: 40.71 (sd: 11.11)
NPRS	mdn: 5 (min-max: 0–8)	mdn: 6 (min-max: 1–9)	*p* = 0.11	mdn: 5 (min-max: 0–9)

*Mdn* median value, *NPRS* Numeric Pain Rating Scale, *PSEQ* Pain Self-Efficacy Scale, *PIT* Prone Instability Test, *RMDQ* Roland Morris Disability Questionnaire, *ROM* Range Of Motion, *SD* standard deviation, *SLR* Straight Leg Raise, * indicates a significant difference (*p* ≤ 0.05).

MC was present in 60 (53.6%) of the participants; a total of 33 (29.5%) had MC1, and 27 (24.1%) of the participants presented MC2 on MRI. MC3 was not seen in any of the participants. The comparison of clinical tests between the two groups showed that the largest difference was observed in reported pain during lumbar spine flexion, 49 (81.7%) in the MC group versus 33 (63.5%) in the no MC group (*p* = 0.03). No significant differences of clinical tests and questionnaires were observed between MC1 and MC2 (Table 2).

**Table 2:** Comparison of clinical tests and questionnaires for patients with MC type 1 or 2

	MC type 1 *n = 33*	MC type 2 *n = 27*	Difference
Positive PIT	24 (72.7%)	21 (77.8%)	*p* = 0.65
Positive SLR	5 (15.6%)	7 (26.9%)	*p* = 0.29
Pain in flexion	27 (81.8%)	22 (81.5%)	*p* = 0.97
Pain in extension	24 (72.7%)	22 (81.5%)	*p* = 0.43
Limited ROM flexion	25 (75.8%)	24 (88.9%)	*p* = 0.19
Limited ROM extension	26 (78.8%)	23 (85.2%)	*p* = 0.52
RMDQ	x̄: 11.00 (sd: 5.25)	x̄: 10.93 (sd: 5.58)	*p* = 0.96
PSEQ	x̄: 41.73 (sd: 10.73)	x̄: 43.89 (sd: 9.66)	*p* = 0.42
NPRS	mdn: 5 (min-max: 0–7)	mdn: 5 (min-max: 0–8)	p = 0.65

*Mdn* median value, *NPRS* Numeric Pain Rating Scale, *PSEQ* Pain Self-Efficacy Scale, *PIT* Prone Instability Test, *ROM* Range Of Motion, *RMDQ* Roland Morris Disability Questionnaire, *SD* Standard Deviation, *SLR* Straight Leg Raise x̄ = mean; * indicates a significant difference (*p* ≤ 0.05)

Univariable logistic regression yielded significant results only for predicting the presence of MC by pain during lumbar spine flexion (Table 3).

**Table 3:** Univariable logistic regression on the presence of MC for clinical tests and questionnaires

	Odds Ratio	95% Confidential Interval	*p*-value	Nagelkerke R^2^
Pain in flexion	2.57	1.08–6.08	0.03*	0.055
Pain in extension	1.89	0.83–4.31	0.13	0.028
Limited ROM flexion	1.64	0.67–4.02	0.28	0.014
Limited ROM extension	2.16	0.90–5.18	0.08	0.036
SLR	0.64	0.27–1.54	0.32	0.012
PIT	1.88	0.84–4.21	0.13	0.028
RMDQ	1.01	0.47–2.16	0.99	0.000
PSEQ	0.51	0.79–3.53	0.18	0.021
NPRS LBP	0.79	0.37–1.72	0.56	0.004

*P-values below assumed a-level of 0.05 for statistical significance, *ROM* range of motion, *SLR* straight leg raise, *PIT* prone instability test, *RMDQ* Roland Morris Disability Questionnaire, *NPRS* Numeric Pain Rating Scale, *PSEQ* Pain Self-Efficacy Scale. ROM was considered ‘limited’ or ‘not limited’ by the assessor at first assessment compared to the time frame before LBP became obvious. Analyses were unadjusted for age and gender

The pain during flexion movements resulted in an odds ratio (OR) of 2.57 (95% CI: 1.08–6.08) for predicting the presence of MC. The Nagelkerke R^2^ measure ranged between 0.00 and 0.06 for all variables tested in the univariable models. Adjusting the univariable analyses for age and gender did not alter the results.

In Table 4, the sensitivity and specificity, positive predictive (PPV) and negative predictive values (NPV) for each single clinical test was calculated, and multi-test regimen are shown. The model showed, for any of the combinations in the multi-test regimen, a low AUC score, ranging from 0.41 to 0.53. The highest sensitivity and specificity was obtained by ≥6 out of 9 tests (61 and 53%) and AUC of 0.53.

**Table 4:** Diagnostic value of clinical tests and questionnaires for Modic changes

Multi-test regimens	Sensitivity	Specificity	PPV	NPV	LR+	LR–	AUC
0 out of 9 tests	1(0.00–0.10)	100(0.9–1.0)	1(0.1–1.0)	46(37.3–56.5)	NA	0.98(0.95–1.01)	0.421
≥1 positive out of 9 tests	99(94.4–99.9)	1(0.00–8.5)	53(43.4–62.6)	0	0	0	0.411
≥2 positive out of 9 tests	98(89.3–98.3)	7(2.4–19.4)	55(45.2–64.6)	8(2.98–9.89)	1.06(0.97–1.15)	0.21(0.01–2.37)	0.445
≥3 positive out of 9 tests	89(81.6–94.0)	13(6.0–26.0)	55(44.7–64.8)	58(28.5–83.5)	1.05(0.92–1.20)	0.61(0.20–1.82)	0.435
≥4 positive out of 9 tests	91(80.8–96.8)	26(15.9–41.2)	59(48.4–69.0)	73(48.5–89.8)	1.25(1.04–1.50)	0.30(0.12–0.79)	0.515
≥5 positive out of 9 tests	80(67.2–81.7)	32(20.7–47.2)	57(46.4–68.4)	58(39.1–75.9)	1.18(0.94–1.49)	0.61(0.34–1.09)	0.488
≥6 positive out of 9 tests	61(48.1–73.6)	52(37.7–65.7)	59(46.4–71.6)	54(39.4–67.9)	1.28(0.90–1.81)	0.73(0.51–1.05)	0.530
≥7 positive out of 9 tests	33(22.0–46.7)	71(56.7–82.4)	57(39.5–73.2)	48(36.6–59.6)	1.15(0.66–.2.01)	0.93(0.77–1.13)	0.500
≥8 positive out of 9 tests	10(4.1–21.1)	90(82.7–94.7)	54(24.5–81.1)	46(36.6–56.6)	1.04(0.33–3.21	0.99(0.91–1.08)	0.333
9 positive out of 9 tests	3(0.01–0.25)	1(0.91–1)	100(0.20–1)	47(37.7–56.9)	Na	96.6(92.2–1.01)	NA
Single tests							
Flexion pain	60 (48.3–70.4)	63 (43.9–80.0)	82 (64.0–90.5)	37 (23.6–51.0)	1.63 (0.99–2.69)	0.64 (0.43–0.93)	0.59
Extension pain	58 (46.6–69.2)	58 (39.2–74.5)	77 (63.9–86.6)	37 (23.5–51.0)	1.37 (0.88–2.13)	0.73 (0.49–1.07)	0.57
ROM flexion	56 (45.3–66.9)	56 (34.9–75.6)	82 (69.6–90.5)	27 (15.6–41.0)	1.28 (0.79–2.07)	0.78 (0.51–1.19)	0.54
ROM extension	58 (47.1–69.0)	61 (40.6–78.5)	82 (69.6–90.5)	33 (20.3–47.1)	1.48 (0.91–2.44)	0.69 (0.46–1.01)	0.57
SLR	44 (25.5–64.7)	45 (33.7–55.9)	21 (11.2–33.4)	71 (56.9–82.9)	0.80 (0.50–1.28)	1.25 (0.82–1.89)	0.48
PIT	58 (46.6–69.6)	57 (39.4–73.7)	75 (62.1–85.3)	39 (25.3–53.0)	1.36 (0.89–2.09)	0.73 (0.49–1.07)	0.57
RDMQ	40 (27.8–53.4)	60 (45.1–72.6)	53 (38.0–68.0)	46 (34.1–58.8)	0.99 (0.63–1.55)	1.00 (0.79–1.27)	0.61
NPRS	57 (43.2–69.1)	54 (39.5–67.5)	59 (44.9–71.1)	52 (37.9–65.4)	1.18 (0.75–1.88)	0.99 (0.70–1.14)	0.53
PSEQ	45 (32.3–58.3)	42 (29.0–56.7)	47 (34.1–60.9)	40 (27.0–54.0)	0.78 (0.54–1.22)	1.33 (0.98–1.72)	0.44

*AUC* Area under the curve, *LR+* Likelihood ratio positive, *LR-* Likelihood ratio negative, *NA* Not Applicable, *NPV* Negative Predictive Value, *NPRS* Numeric Pain Rating Scale cut-off point ≥4, *PPV* Positive Predicted Value, *PSEQ* Pain Self-Efficacy Scale cut-off point ≥42, *PIT* Prone Instability Test, *RMDQ* Roland Morris Disability Questionnaire cut-off point ≥10, ROM range of motion, *SLR* Straight Leg Raise

In the single-test analyses, the AUC was higher, ranging from 0.48 (straight leg raise) to 0.59 (pain during flexion movement). The sensitivity for assessment of present MC was the highest for pain during flexion movement, 60% (95% CI: 48.3–70.4%), which also showed the highest specificity: 63% (95% CI: 43.9–80.0%).

## Discussion

The aim of this explorative study was to assess the predictive value of clinical test and questionnaires to detect MC in a population of active servicemen of the Netherlands Armed Forces with persistent LBP.

The main finding of this study was that, in general, the clinical tests and questionnaires that were tested did not perform very well in predicting the presence of MC accurately in Dutch military personnel. Although pain on movement, including in the PIT, or limited movement was more prevalent in people with MC, only pain during. flexion was significantly associated with MC. Further, none of the results from the multi-test regimen showed a convincing diagnostic ability to identify people with MC.

These results are in line with previous studies that have reported positive associations between the presence of MC and single clinical findings such as pain on movement [10, 11], inflammatory pain pattern [13], and segmental instability [15, 16]. The theoretical rationale for these factors being associated with MC is that the vertebral endplates adjacent to MC, especially of MC type 1, contain immunoreactive nerve endings, and it has been reported that an increased number of tumor necrosis factor–immunoreactive nerve cells and fibers are present in endplates that have MC [29]. Pain on movement may therefore originate from loading the damaged endplates where MC are present. Another explanation is that MC is a proxy for discogenic pain, because MC are most often seen in relation to moderate to severe disc degeneration [12, 14] and that immunoreactive nerves have been shown to be present in degenerative discs [30].

One reason for finding only small differences in pain between patients with and without MC on movement may be that our population consisted of servicemen who have a high pain threshold or motivation to continue physical tasks due to the perspective of continuing their military career despite having LBP [31].Another reason for finding modest differences in our analysis could be that our patient sample consisted of both patients with leg pain and those without. People with leg pain may report pain on movement due to the disc herniation or extrusion causing the problem. Because of the limited number of included patients in the study, it was not possible to perform meaningful stratification based on the presence of radiculopathy on SLR.

The population of military personnel included in this study may seem a selected group of people, and the generalizability of the results from our study may seem restricted by the fact that our population consisted of servicemen. However, the study population is comparable to that previously studied by others regarding NPRS and RMDQ; only mean age was slightly lower, and only a few females were included in our cohort. These factors may have introduced selection bias. However, because sex has been reported to have no impact on the presence of MC [3], we do not believe that this could have influenced the results. The analysis was adjusted for age; however, it did not show any differences compared to unadjusted models.

### Limitations

This study has several limitations. First, this study was performed based on a non-general population, including a selective group of people, i.e., military men and women only. This relatively young population may have a higher risk of developing degenerative spinal changes, including MC, or other reasons for LBP, because the participants in all military ranks endure a high physical workload [10, 31]. Although this may have influenced the results, as mentioned earlier, the population’s characteristics can be compared to that of other studies. In our view, this higher physical workload is only seen in the enlisted personnel, although noncommissioned officers and commissioned officers, who endure high physical workload less frequently, were also included.

Second, the development of a multivariable prediction model requires a large sample to assess all possible factors accurately. In this study, due to its retrospective nature, we were limited to 112 participants with accurate patient files and available MR images, which reduced the possible exploration of variables, and we could therefore not do a reliable multivariable model due to the limited number of individuals. This may indicate the need for future research, including larger study samples.

Another issue are the possible inconsistencies in conducting and interpreting the clinical tests by the two examiners, which might have a negative impact on the diagnostic accuracy. However, we find that the internal consistency in this study was appropriate as the tests were conducted and interpreted by two experienced manual therapists, who have had similar training and who have worked with each other for the last 25 years.

For daily clinical practice, it would be desirable for any symptoms or signs to be demonstrable so that diagnose of the presence of MC could be accomplished in a reliable manner. However, it seems that the contrast in clinical signs between patients with MC and without MC seems negligible. MC is proposed to be classified as a separate clinical subgroup among patients with nonspecific low-back pain (NSLBP) [10]. However, at this stage, MRI is the best way to assess the presence and classification of MC reliably.

## Conclusion

The results of our study suggest that the only clinical test associated with the presence of MC was pain during flexion movement patterns. Furthermore, no models of multi-test regimens reached acceptable diagnostic accuracy. Patients with LBP and MC do therefore not appear very different from other patients with LBP and based on the clinical tests and questionnaires investigated in this study they are therefore not likely to form a specific subgroup of LBP. However, due to the sample size we only included a limited number of clinical variables and it is possible that a larger study sample allowing for more variables would show a different result.

## Abbreviations

AUC: Area under the curve
CCMO: Centrale Commissie Mensgebonden Onderzoek
CI: Confidence interval
DGO: Defensie Gezondheidszorg Organisatie
ICC: Intra class correlation
LBP: Low back Pain
MC: Modic change
MRI: Magnetic resonance imaging
MRC: Military rehabilitation centre aardenburg
NPRS: Numeric pain rating scale
NPV: Negative predictive value
PSEQ: Pain self-efficacy questionnaire
PIT: Prone instability test
PPV: Positive predictive value
PSFS: Patient specific functional scale
RMDQ: Roland Morris disability questionnaire
SLR: Straight leg raising
STROBE: Strengthening the Reporting of observational studies in epidemiology
